# Efficacy of a High-Protein Diet to Lower Glycemic Levels in Type 2 Diabetes Mellitus: A Systematic Review

**DOI:** 10.3390/ijms252010959

**Published:** 2024-10-11

**Authors:** María Nelly Flores-Hernández, Hilda Martínez-Coria, Héctor E. López-Valdés, Marcela Arteaga-Silva, Isabel Arrieta-Cruz, Roger Gutiérrez-Juárez

**Affiliations:** 1 Departamento de Ciencias Biomédicas, Escuela de Medicina, Facultad de Estudios Superiores Zaragoza, Universidad Nacional Autónoma de México, Mexico City 09230, Mexico; nellyflores0512@gmail.com; 2 Departamento de Fisiología, Facultad de Medicina, Universidad Nacional Autónoma de México, Mexico City 04360, Mexico; hildamcoria@gmail.com (H.M.-C.); helopezv@gmail.com (H.E.L.-V.); 3 Departamento de Biología de la Reproducción, Universidad Autónoma Metropolitana-Iztapalapa, Mexico City 09340, Mexico; arteaga1967@hotmail.com; 4 Departamento de Investigación Básica, División de Investigación, Instituto Nacional de Geriatría, Secretaría de Salud, Mexico City 10200, Mexico; iarrieta@inger.gob.mx

**Keywords:** diabetes mellitus, type II, diet, high protein, hyperglycemias, glycated hemoglobin A, amino acids, branched chain

## Abstract

Diabetes is a metabolic disease with a high worldwide prevalence and an important factor in mortality and disability in the population. Complications can be reduced or prevented with lifestyle changes in physical activity, dietary habits, and smoking cessation. High-protein diets (HPDs, >30% or >1.0 g/Kg/day) decrease hyperglycemia in part due to their content of branched-chain amino acids (BCAAs), mainly leucine. Leucine (and other BCAAs) improve glucose metabolism by directly signaling in the medio-basal hypothalamus (MBH), increasing liver insulin sensitivity. To determine the effectiveness of an HPD to lower hyperglycemia, we analyzed the results of published clinical studies focusing on the levels of fasting plasma glucose and/or glycosylated hemoglobin (HbA1c) in patients with type 2 diabetes mellitus (T2DM). We carried out a systematic search for clinical studies using HPDs. We searched five databases (Scopus, Web of Science, PubMed, Epistemonikos, and Cochrane), collecting 179 articles and finally selecting 8 articles to analyze their results. In conclusion, HPDs are an effective alternative to reduce hyperglycemia in patients with T2DM, especially so-called Paleolithic diets, due to their higher-quality protein from animal and vegetal sources and their exclusion of grains, dairy products, salt, refined fats, and added sugars.

## 1. Introduction

Diabetes mellitus is a general term for heterogeneous metabolic alterations, with the main finding being an increase of glucose concentration in the blood, known as hyperglycemia, that occurs chronically and appears to be a progressive loss of secretion of β-cell insulin, usually in the context of insulin resistance [1,2]. When insulin resistance exists, the body does not respond to it. Therefore, glucose levels rise, causing a greater release of insulin as compensation, which can wear down the pancreas, causing that over time, the body produces less and less insulin and further increases hyperglycemia levels [3]. According to recent data from the International Diabetes Federation (IDF), 537 million adults worldwide had diabetes in 2021, and this number is predicted to rise to 643 million by 2030 [4]. Furthermore, in 2021, diabetes was directly responsible for 6.7 million deaths. This increase of glucose concentration in the blood is attributed to little or no physical activity and the consumption of Western-style diets containing high amounts of saturated fatty acids, salt, and refined sugar [5], all of which represent risk factors for the onset of metabolic diseases such as obesity and type 2 diabetes mellitus (T2DM). In this way, the American Diabetes Association (ADA) and the US Academy of Nutrition and Dietetics recommended lifestyle modifications to improve medical outcomes and quality of life for adults with prediabetes or T2DM, with a high cost/benefit ratio. Among the actions taken, a control system was developed that includes diabetes self-management education, nutritional therapy support, physical activity, smoking cessation counseling, and psychosocial care [6,7]. Current ADA dietetic recommendations for patients with diabetes are as follows: non-starchy vegetables as the basis for dishes; lean proteins and plant-based sources of protein; quality carbohydrates such as starchy vegetables, fruits, whole grains, and low-fat milk; less added sugar; healthy fats; less processed foods; and water or zero-calorie beverages [8]. Therefore, the nutritional management of patients with diabetes, in addition to appropriate hypoglycemic therapy, can be effective in helping to normalize hyperglycemia [3,9]. To assess glycemic control, the measurement of glycated hemoglobin (HbA1c) is used as a key tool since it has a strong predictive value for diabetes complications. Additionally, HbA1c levels reflect the average blood glucose over 3 months prior to the measurement. Continuous monitoring of blood glucose concentrations also plays an important role in evaluating the efficacy and safety of T2DM treatment [10,11].

On the other hand, the risk factors associated with the development of T2DM, such as non-modifiable factors (aging, genetics) and modifiable factors (hypercaloric diet, sedentary lifestyle, stress, alcoholism, smoking, changes in sleep patterns, etc.), are well known to induce epigenetic changes at different levels, including the following: DNA methylation, histone modification, non-coding RNA-mediated regulation, affecting the expression of genes associated with the control of insulin secretion and sensitivity, glucose metabolism, inflammatory control mechanisms, and adipose tissue functionality. This contributes to the progression and poor prognosis of T2DM as well as its association with other cardiovascular diseases [12,13,14,15]. Therefore, the inclusion of functional foods in the diet could help to improve the physiological disturbances observed in T2DM by means of reverting epigenetic changes that originally caused them, thus potentially reducing the deleterious effects of risk factors associated with T2DM.

It is known that amino acids ingested in the diet favor an adequate nutritional status due to stimulation of protein synthesis [16]. It has also been known for decades that dietary protein content exerts powerful effects on insulin sensitivity, glucose metabolism, and body adiposity [17]. It has been shown in seminal studies that an increase in the protein content of the diet to ≥30% of total energy, or ≥1.2 g/Kg/day, at the expense of carbohydrates can improve glycemic control [18] in a way comparable to some oral agents and without significantly affecting renal function [19]. This effect is caused by several factors, including the following: (1) less insulin (endogenous or exogenous) is needed for protein metabolism [20,21]; (2) proteins cause greater satiety [22]; and (3) amino acids can increase insulin sensitivity and secretion [23]. Regarding the third point, the effect is due specifically to the presence of branched-chain amino acids (BCAAs), mainly leucine (Leu) [24,25,26]. As an additional effect, high protein intake decreases the risk of obesity since it causes greater satiety [22] and increases resting caloric expenditure secondary to decreased glucose oxidation in skeletal muscle tissue [20,21].

Earlier studies showed that the intravenous administration of BCAAs caused a decrease in glucose levels, with a concomitant increase of circulating insulin [27]. Further studies disclosed that leucine was the main BCAA involved in the improvement of glycemic control [28] and that high-protein diets reduced the risk of T2DM [29]. One mechanism by which dietary protein improves insulin action and glucose metabolism is through the sensing of nutrient abundance in the medio-basal hypothalamus (MBH), responsible for the control of food intake and energy metabolism [30]. Leucine, the most abundant BCAA, has also been the most studied and is known to act as a nutrient signaling molecule in the brain. It can directly access the MBH as it bypasses the blood–brain barrier (BBB) through a permeable microvasculature with endothelial cells fenestrated specifically in the arcuate nucleus (ARC) of the MBH. In the ARC, leucine is metabolically converted to acetyl-CoA and then activated to malonyl-CoA, a precursor for the synthesis of fatty acids. Leucine would finally produce oleoyl-CoA, a long-chain acyl CoA (LCAC) that causes the activation of hypothalamic ATP-sensitive potassium (K_ATP_) channels, generating a signal that reaches the liver through the vagus nerve. Vagal stimulation to the liver produces a glucoregulatory response by reducing hepatic glucose production through the inhibition of gluconeogenesis and glycogenolysis, ultimately leading to a decrease of circulating glucose levels [30,31]. Interestingly, studies in rodents showed that inactivation of these regulatory mechanisms in the hypothalamus leads to the development of postprandial hyperglycemia and hyperinsulinemia compared to intact animals [30]. Another glucoregulatory mechanism of HPDs involves the incretin effect, in which BCAAs cause an increase in gut hormones such as glucagon-like peptide-1 (GLP-1) and glucose-dependent insulinotropic peptide (GIP). These peptides improve the functional balance of pancreatic α and β cells by decreasing the production of glucagon and increasing the release of insulin, respectively. At the same time, these entero-hormones can also stimulate satiety [32,33].

Ile and Val share the same metabolic pathway as Leu, at least down to acetyl-CoA, and they should have a similar effect in reducing glucose levels, which, in fact, they do [24]. However, BCAAs have been found to exert metabolically negative effects, which have been attributed to Ile and Val, not to Leu [34,35]. In a recent study, reducing isoleucine or valine rapidly restores metabolic health to diet-induced obese mice, while variation in dietary isoleucine levels helps explain body mass index differences in humans [35]. In an even more recent study, the dietary restriction of isoleucine increases healthspan and lifespan in mice [36]. Thus, some of these observations may help to explain why the intake of a diet with a high content of Ile and Val can cause an increase in BMI, increasing the risk of metabolic diseases [34,37]. Thus, depending on the content of each AA in the food, they could cause different responses. However, they are always ingested together, generally in a ratio of approximately 2.2:1.0:1.6 Leu:Ile:Val [37]. Accordingly, leucine, (BCAA found in greater proportion) is the one that will determine the effect of protein ingestion.

Although there is enough information to support the glucoregulatory properties of Leu, there are also studies that link a high circulating concentration of BCAAs with an increased risk of insulin resistance, a high BMI, T2DM, and other metabolic diseases [38,39]. Thus, the goal of this work was to determine the efficacy of HPDs to reduce blood glucose in patients with T2DM based on an analysis of the results obtained in different clinical studies that test high-protein diets and in which fasting serum glucose and glycated hemoglobin (HbA1c) values were used as monitoring parameters to generate new alternatives that contribute to the treatment of T2DM.

## 2. Materials and Methods

### 2.1. PICO Methodology

The methodology was delimited by the PICO structure; the established population was adults with T2DM, the intervention used was a high-protein diet compared to the conventional diet for diabetes, and the expected outcomes were decreases of either fasting blood glucose or glycosylated hemoglobin.

### 2.2. Search Algorithm

To define the structure of the search algorithm, a search was made for concepts derived from the following terms: type 2 diabetes mellitus, measurement of blood glucose and glycosylated hemoglobin, and high-protein diet. The search was carried out with different combinations of terms and Boolean operators until the algorithm was configured to show enough records in each database. Accordingly, the following final algorithm was used: (“Hyperglycemia” OR “Diabetes Mellitus type 2” OR “Insulin resistance”) AND (“High protein diet”) AND (“Blood Glucose” OR “Glycated Hemoglobin A”).

### 2.3. Databases

**Figure 1:**
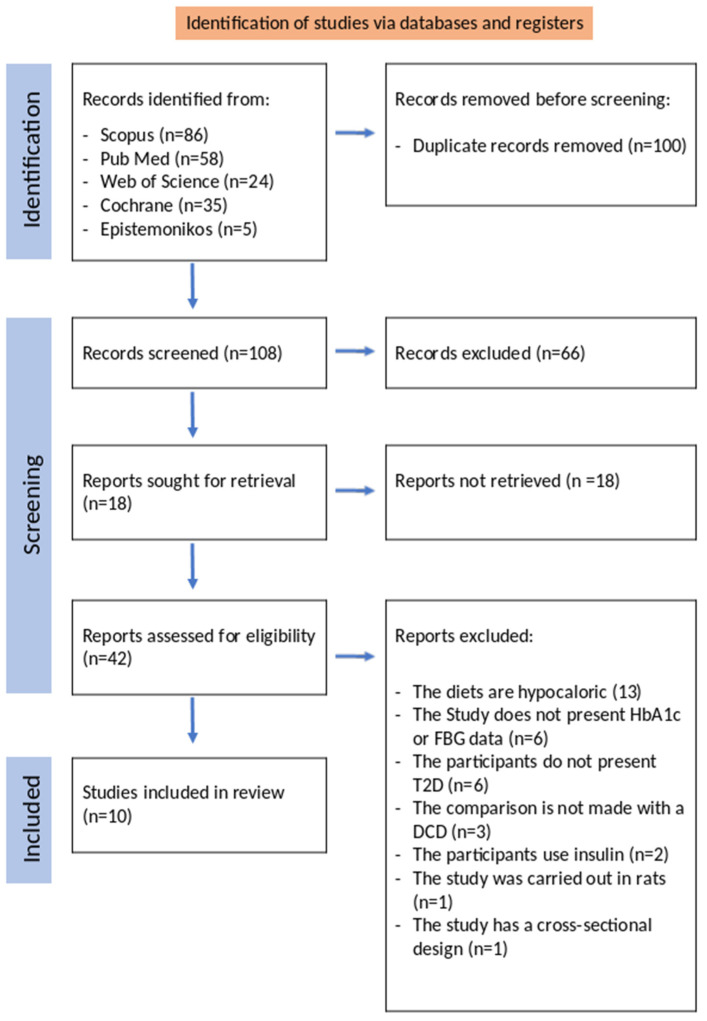
PRISMA 2020 flow diagram showing the selection method for the articles considered for review.

The search was carried out on the following 5 databases: Scopus, Web of Science, PubMed, Epistemonikos, and Cochrane, using the algorithm in Section 2.2 on each of the databases. The number of articles found in each database and the way in which the records were selected is described in Figure 1. To select the articles, both inclusion and exclusion criteria were established to reduce bias and thus avoid possible confounding factors. These criteria are summarized in Table 1.

### 2.4. Dates of Search for Document Selection

For this work, overall, we searched articles for analysis from the year 1974 to March 2023 in the various databases in Section 2.2 above.

### 2.5. PRISMA Standards

The reporting of this systematic review was guided by the standards of the Preferred Reporting Items for Systematic Review and Meta-Analysis (PRISMA) Statement [40] (Figure 1).

### 2.6. Assessment of Risk of Bias (ROB)

Assessment of the ROB for the studies selected in this review was performed using the Cochrane’s Review Manager (RevMan) tool [41].

## 3. Results

The results obtained are summarized in Table 2. In this table, the data from each selected article, some of its most relevant characteristics, and the necessary results to carry out the analysis were placed. On the other hand, the articles excluded from the analysis [42,43,44,45,46,47,48,49,50,51,52,53,54,55,56,57,58,59,60,61,62,63,64,65,66,67,68,69,70,71] were placed in Supplementary Table S1. In total, eight articles were reviewed, of which two did not report FBG data and one did not report HbA1c data; even with this, a decrease of FBG was observed in four studies, and decrease of HbA1c was observed in five studies. Among the studies in which a decrease in FBG was not observed, a decrease in HbA1c was observed instead. Only one study did not see a decrease in blood glucose at the end of the study and did not report HbA1c results. The total number of participants was 417, including men and women, but without specifying how many of each. In most of the studies selected for analysis, the information about the sex of the participants was indicated. However, the authors of the original studies did not perform analysis of the data stratified by sex. In the studies chosen for this analysis, no major differences in body adiposity either between treatments or at the beginning and the end of the different dietary regimes was observed (Supplementary Table S2). Supplementary Table S2 summarizes the changes in body adiposity, either measured by BMI or simply by body weight, indicating a minor or no role of body adiposity on the glycemic changes caused by a HP diet. In fact, in the table, we see only minuscule changes in BMI, either between treatment groups or by comparing BMI at the beginning and the end of the dietary treatments. In some cases, there was a reduction of BMI, but it was of the same magnitude in both treatment groups, thus ruling out body adiposity as a confounding factor.

In one study [74], the authors attribute the lowering of blood glucose and HbA1c to which ω-3 fatty acids may enhance outcomes with the use of the high-protein diet. These results present a greater effect on the reduc63tion in HbA1c than the diet in which the proportion of proteins is simply increased (*p* = 0.03), or with the normal diet supplemented only with ω-3 (*p* = 0.01), at least for 12 weeks. In another study [72], no results are presented on the significant decrease in fasting blood glucose; however, it does present results on another important variable: it was observed that in this study, the HP diet reduced postprandial plasma glucose concentrations, which is also beneficial. The determination of postprandial blood glucose is important to achieve adequate glycemic control, and its alteration can modify glucose homeostasis [82]. The authors also highlight that this HP diet, which reduced postprandial blood glucose, contains a higher amount of vitamin D and calcium. In three of the studies, the safety of protein consumption was investigated, since it has been reported that a high intake of these macronutrients can cause albuminuria or kidney damage. However, the results show that kidney function was not affected. On the contrary, in the study by Pomerleau (1993), an improvement was observed [81].

**Figure 2:**
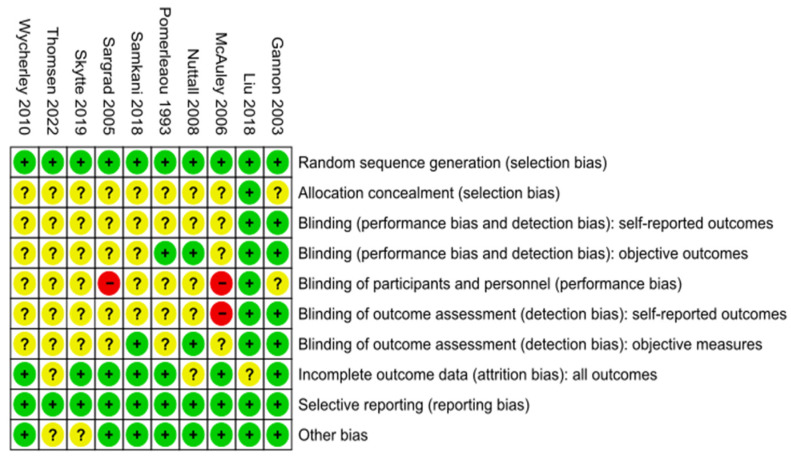
Assessment of risk of bias (ROB) of the included studies performed using the RevMan tool [41,53]. Thomsen 2022 [42]; Skytte 2019 [43]; Liu 20018 [44]; Samkani 2018 [45]; Wycherley 2010 [46]; Nuttal 2008 [47]; McAuley 2006 [48]; Sargrad 2005 [49]; Gannon 2003 [50]; Pomerleaou 1993 [51].

When performing a more in-depth study of the results, the behavior of glucose parameters when an HPD is ingested shows improvement compared to a DCD, even without differences in fasting blood glucose and glycosylated hemoglobin. In the study by Samkani et al. [75], glycemia decreases with an HPD by 18% and by 15% after breakfast and lunch, respectively. In the same way, compared with a DCD, an HPD reduced total glucose AUC by 14% and net glucose AUC by 121%. Insulin also showed improvement with an HPD compared with a DCD, reducing the peak serum insulin concentration by 24% and 21% after ingestion of breakfast and lunch, respectively [72]. In the same study, an HPD reduced total insulin AUC by 22% and net insulin AUC by 33%, confirming one more benefit of HPDs [72]. We carried out an assessment of the risk of bias (ROB) for the included studies (Figure 2). A risk of bias (ROB) assessment (also called “quality assessment”) is helpful to establish transparency of evidence synthesis results and findings [83]. The goal of an ROB is to eliminate bias in a study’s findings, such as biases in the results or conclusions, including design flaws that may raise questions about the validity of the findings.

## 4. Discussion

The studies analyzed in this review confirm the efficacy of an HPD in improving glucose metabolism in T2DM patients compared with a conventional diet (DCD). This improvement was mainly reflected by a decrease in fasting plasma glucose and/or glycosylated hemoglobin. In seven of the eight studies reviewed, this reduction was observed in at least one of the two parameters, and in only one study was no reduction reported. However, the latter study did not measure HbA1c, so it cannot be concluded that an HPD did not have a stable effect on blood glucose levels. Although we observed a decrease in glycemia after the intervention with a DCD, the effect with an HPD was much more pronounced. The possible reasons for this phenomenon could be the following: (a) Both diets, a DCD and an HPD, were formulated by nutritionists using foods with a higher content of beneficial components not commonly found in the daily diet of the general population (such as fiber) and a reduction of harmful foods (such as processed foods, high salt content, or high glycemic index); (b) a common effect in clinical trials, known as the Hawthorne effect, in which participants increase their attention to their disease and make involuntary and subtle changes in their activities because they feel monitored during the studies [84].

As mentioned above, the BCAAs in dietary proteins are partially responsible for the reduction of hyperglycemia, so it would be convenient to consume protein foods rich in BCAAs. The content of BCAAs in foods depends on their origin, whether animal or plant, and depending on the amount and type of BCAAs, they can produce different intensities in the metabolic response, but both sources are essential [85]. Animal proteins have a higher nutritional value due to their higher content of BCAAs. In addition, they have a high protein density with a lower calorie content, which promotes a greater benefit than vegetable proteins. On the other hand, vegetable proteins delay gastric emptying and have a high fiber content. Although both types of protein control appetite and satiety, plant proteins not only contain much lower levels of BCAAs but are also less digestible compared to animal protein [18]. In addition, the foods included in an HPD should be of high quality, which means that they must be derived primarily from lean animal sources [40]. Importantly, the types of foods used in the selected studies were mostly from low-fat sources, such as lean fish; low-fat milk, yogurt, and cheese; and high-protein supplements in the form of powders and bars. Other high-quality meat options include free-range cattle and birds and their eggs; blue fish, such as salmon; and rabbit. In the case of blue fish, there is an additional metabolic health benefit because their high content of unsaturated fatty acids, especially omega-3, are precursors of anti-inflammatory molecules that improve insulin sensitivity and may be helpful in reducing the risk of T2DM [86].

Processed red meat has potentially harmful components when consumed in large amounts over a long period of time. These include saturated fatty acids that promote inflammation and insulin resistance; large amounts of sodium, nitrates, or nitrites used in processing; trimethylamine oxide; and endocrine disruptors that interfere with human physiological systems [87]. The deterioration of meat quality is a result of the increased demand for food due to the accelerated growth of the world’s population, which has favored the mass production of meat from industrialized farms. This meat comes from animals that grow at a faster rate and lack physical activity, causing meat to contain more saturated fats and other harmful elements. In summary, the type of diet chosen must offer the greatest benefits with the least negative effects.

Currently, there are several types of diets that differ in terms of the amount of food consumed, the type of food (macronutrient composition), or the timing of meals. All these factors can affect the metabolic health of patients [88]. The reason we focus on high-protein diets and the so-called Paleolithic diet is that consumption of these diets improves glycemic control and several cardiovascular risk factors compared to a DCD [89,90]. Such diets have been called “Paleolithic” because of their similarity to the diet consumed by humans in the Paleolithic era. According to research by anthropologists and paleontologists, our human ancestors consumed a diet of which more than 50% was meat and the rest was plant foods, especially fruits and nuts. This diet included lean, healthy meat from wild animals raised in an outdoor environment. This regime changed with the development of agriculture, when the amount of meat consumed decreased significantly. In contrast, foods of plant origin increased and now constitute up to 90% of the diet [89]. As a result, humans began to replace red meat with vegetables, grains, and industrialized meats, causing a wave of metabolic diseases not seen in the Paleolithic era. These diseases included diabetes, cardiovascular disease, hypertension, and even some types of cancer [90]. Interestingly, some modern human groups that still follow a Paleolithic-like diet do not suffer from any of today’s metabolic diseases [91].

Foods consumed in this diet include most lean meats, fish, and, to a lesser extent, shellfish, fruits, vegetables, roots, eggs, and nuts, and should exclude grains, dairy products, salt, refined fats, and added sugars [87]. The foods used in the studies included in this review contained protein from lean sources, consistent with the need to use high-quality protein, as well as low-glycemic fruits and nuts, which are components of the Paleolithic-type diet. The Paleolithic-type diet also contains a minimal amount of grains, consistent with the postulate that humans have an insufficient adaptation of the leptin system to grains [92]. The carbohydrates used in the studies are small, in the form of high-fiber, low-glycemic-index grains, bread known commercially as “light bread,” low-starch vegetables, and low-glycemic-index fruits. The foods that are not consistent with the Paleolithic-type diet are dairy products with their derivatives and some processed foods, although it has been observed that the consumption of dairy products as a source rich in BCAAs has been associated with a lower incidence of T2DM [18]. In the case of processed foods, a greater benefit may be achieved by eliminating them from the diet. Based on the above information, the so-called Paleolithic diet is likely to be one of the best options for supporting the treatment of T2DM.

As mentioned previously, to achieve the expected response with an HPD, it is necessary that dietary plans are designed by expert nutritionists, while patients are closely monitored to achieve the most robust adherence to the diet. In addition to adherence to diets, another factor that may negatively influence compliance with recommended dietary regimes is limited access to sources with a good content of high-quality protein, usually of animal origin, which may be expensive for some segments of the population. This is especially important given the chronic pattern of recommended intake of these diets. In addition, the long-term safety of HPD intake should be considered, especially in a carbohydrate-restricted regimen, as it can lead to hypoglycemia in people with T2DM, especially those on pharmacological treatments [89]. High-protein regimens have also been suggested to increase the risk of renal dysfunction [93,94]. However, in three of the studies reviewed, assessment of renal function showed that there was no detectable damage to the kidneys, and patients even showed a slight improvement. It is important to note that the selected studies were conducted only in individuals without advanced or severe kidney disease [93]. It has been said that HPDs increase the risk of fractures and osteoporosis by accelerating bone resorption and the urinary excretion of calcium, but it has been seen that protein intake is negatively correlated with bone loss and is not significantly different from low-protein diets [68]. It should be kept in mind that the metabolic effects of the tested diets are not permanent, as it has been shown that the metabolic benefits slowly decrease when the diets are discontinued [67]. Table 3 displays a summary of potentially beneficial versus detrimental effects of the consumption of an HPD.

Protein-derived leucine is known to act as a nutrient-signaling molecule in the brain [30,31]. It can directly access the MBH as it bypasses the blood–brain barrier (BBB), specifically in the arcuate nucleus (ARC). In the ARC, leucine is metabolically converted to acetyl-CoA and then activated to malonyl-CoA, a precursor for the synthesis of fatty acids. Leucine would finally produce oleoyl-CoA, a long-chain acyl CoA (LCAC) that then activates hypothalamic ATP-sensitive potassium (K_ATP_) channels, generating a signal that is relayed to the liver via the vagus nerve. Vagal efflux to the liver causes a reduction of hepatic glucose production through the inhibition of gluconeogenesis and glycogenolysis, ultimately leading to a decrease of circulating glucose levels [30,31]. Interestingly, studies in rodents showed that inactivation of these regulatory mechanisms in the hypothalamus lead to the development of postprandial hyperglycemia and hyperinsulinemia compared to intact animals [31]. Another glucoregulatory mechanism of HPDs involves the incretin effect, in which BCAAs cause an increase in gut hormones such as glucagon-like peptide-1 (GLP-1) and glucose-dependent insulinotropic peptide (GIP). These peptides improve the functional balance of pancreatic α and β cells by decreasing the production of glucagon and increasing the release of insulin, respectively. At the same time, these entero-hormones can also stimulate satiety [32,33].

Although most studies suggest that BCAAs improve insulin sensitivity, there is some experimental evidence that high circulating levels of BCAAs are associated with insulin resistance and other metabolic disorders [38,39]. However, it is currently unclear whether high levels of BCAAs are a cause or a consequence in the development of these diseases. These studies did not determine the exact mechanism by which BCAAs could cause the mentioned metabolic conditions [38]. Even the authors of the studies themselves mentioned that it cannot be concluded that BCAAs are the cause based solely on the associative metabolic data they found [39]. Another layer of complexity that perhaps would help to clarify the apparently opposing effects of BCA observed is added by a series of recent studies that identified Val and Ile as the BCAA behind some of the adverse effects attributed to BCAAs as a group [34,35].

It is also important to note that many of the studies were conducted in preclinical models, where the results showed an association that was not so clearly observed in clinical studies [38]. Only in case–control studies has an association been observed, but another point that should be emphasized is that most of these observations were made in people who already had some degree of metabolic disorders and associated risk factors [41]. It is therefore necessary to perform longitudinal studies to clarify whether the high levels of BCAAs or their metabolites could have a causal role in the development of insulin resistance.

## 5. Conclusions

In conclusion, a high-protein diet is recommended, in a proportion of 30% of the total energy requirement, or an amount ≥1.1 g/Kg/day in the form of protein, with the aim of reducing blood glucose and favoring the development of a healthier metabolic profile. An HPD can be a very useful strategy for the comprehensive management of T2DM, along with appropriate pharmacological treatment. In particular, a Paleolithic-type diet could be an ideal choice, combining food sources with the lowest content of harmful substances together with a sufficiently high amount of protein that does not compromise renal function.

## Figures and Tables

**Table 1:** Inclusion and exclusion criteria used in the review.

Inclusion Criteria	Exclusion Criteria
- Adults with hyperglycemia - Patients diagnosed with type 2 diabetes mellitus - Reduction to HbA1c levels < 6.5% (<126 mg/dL) - Reduction to HbA1c levels < 7% (<154 mg/dL) - Reduction to HbA1c levels < 8% (<183 mg/dL) - Longitudinal studies - Serum glucose measurement	- Patients who did not remain in the established dietary regimen - Patients with T2DM complications - Pregnant women with T2DM - Patients using insulin - Unstable blood glucose levels - Studies that did not have outpatient control - Measurements made while using drugs that interfere with tests

HbA1c: glycosylated hemoglobin; T2DM: type 2 diabetes mellitus.

**Table 2:** Characteristics of the study populations, the proportions of macronutrients in the diets used, and data of fasting glucose and glycosylated hemoglobin measurements.

Study	Participants	Study Duration	Intervention	Outcome	
				HbA1c	FBG
Thomsen 2022 [72]	72 (Gender NP) Age: 67.0 ± 8.8 years BMI: >25 kg/m^2^	6 weeks	HP: 30% CH, 30% P, 40% F DCD: 50% CH, 17% P, 33% F	HP reduced −0.83 ± 0.38% vs. DCD −0.66 ± 0.37 ^+^	HP reduced −1.7 (−2.7,−0.9) vs. DCD −1.5 (−2.3,−1.0) ^+^
Skytte 2019 [73]	28 (20 male, 8 female) Age: 64 ± 7.7 years BMI: 30.1 ± 5.2 Kg/m^2^	12 weeks	HP: 30% CH; 30% P; 40% F DCD: 50% CH; 17% P; 33% F	HP reduced −6.2 ± 0.8 mmol/mol vs. DCD −0.75 ± 1.0 mmol/mol, (−0.6 ± 0.1% vs. −0.1 ± 0.1%) ^+^	HP reduced −0.71 ± 0.20 mmol/L vs. DCD 0.03 ± 0.23 mmol/L) *
Liu 2018 [74]	122 (Gender NP) Age: 40–60 years BMI: 18.5–23.9 Kg/m^2^	12 weeks	HP: 42% CH, 28% P, 30% F, (PUFA ω-6:ω-3 of 7.0–7.5:1) DCD: 54% CH, 17% P, 29% F, (PUFA ω-6:ω-3 of 7.0–7.5:1)	HP reduced −0.29% (95% CI: −0.43%−0.16 *^₹^* vs. DCD −0.05% (95% CI: −0.19%, −0.08%) NS	HP decreased −0.73 mmol/L (95% CI: −1.13–0.33 mmol/L) *
Samkani 2018 [75]	16 (14 male, 2 female) Age: Me = 65 (43–70) years BMI: 30 ± 4.4 kg/m^2^ HbA1c 6.5% (47 mmol/L) FBG 8.2 ± 2.0 mmol/L	12 months	HP: 31% CH, 29% P, 40% F DCD: 54% CH, 16% P, 30% F	Not reported	No difference
Wycherley 2010 [76]	83 (male, female) Age: 55.0 ± 8.4 years BMI: 35.3 ± 4.5 kg/m^2^ Sedentary	16 weeks	HP: 43% CH; 33% P; 22% F DCD: 53% CH; 19% P; 26% F	HP decreased −1.8 ± 1.6 (from 8.0 ± 1.8 to 6.3 ± 0.9 mmol/L) vs. DCD −1.1 ± 0.6 (from 7.6 ± 1.0 to 6.4 ± 0.7 mmol/L) ^+^	HP decreased −2.5 ± 2.7 (from 9.5 ± 2.9 to 7.0 ± 1.0 mmol/L) vs. DCD −2.2 ± 2.2 (from 9.2 ± 2.7 to 7.1 ± 1.0 mmol/L) ^+^
Nuttall 2008 [77]	8 men Age: 50–67 years BMI: 24–35 Kg/m^2^	5 weeks	HP: 30% CH, 30% P, 40% F	HP −2.2% vs. DCD −1.7% ^¥^	HP −5 mmol/L vs. DCD No Change ^+^
McAuley 2006 [78]	52 (female) Age: 30–70 y BMI: >27 Kg/m^2^ insulin resistance.	12 months	HP: 40% CH with a low glycemic index; 30% P; 30% F (unsaturated); DCD: >55% CH; 15% P; <30% F (<8% SFAs); 25–30g/day of dietary fiber.	Not reported	HP: Initial 5.2 ± 0.5 mmol/L, 1 year 4.9 ± 0.5 mmol/L vs. DCD initial 5.0 ± 0.6 mmol/L, 1 year 4.9 ± 0.5 mmol/L
Sargrad 2005 [79]	12 (3 male, 9 female) Age: 48 ± 5.5 years BMI: 36 ± 3 Kg/m^2^	8 weeks	HP: >proteins; <carbohydrates. DCD: <protein; >carbohydrates.	HP decreased with no significant change from 7.6 ± 0.9% to 6.6 ± 0.5% vs. DCD from 8.2 ± 0.5% to 6.9 ± 0.4% *	HP without significant change of 8.3 ± 1.2 vs. 8.3 ± 1.5 mmol/L vs. DCD decreased from 8.8 ± 0.9 to 7.2 ± 1.0 mmol/L *
Gannon 2003 [80]	12 (10 male, 2 female) without treatment Age: 61 (39–79) years BMI: 31 (22–37) kg/m^2^	5 weeks	HP: 40% CH; 30% P; 30% f (10% MUFAs, 10% PUFAs, 10% SFAs). DCD: 55% CH; 15% P; 30% F (10% MUFAs, 10% PUFAs, 10% SFAs).	HP decreased from 8.1 ± 0.3% to 7.3 ± 0.2% * vs. non-significant DCD from 8.0 ± 0.2% to 7.7 ± 0.3% NS	No difference: 6.3 ± 0.3% mmol/L (114 ± 6 mg/dL)
Pomerleau 1993 [81]	12 (8 male, 4 female) Age: 58 ± 11 years BMI: > 27 kg/m^2^ T2DM with 10 ± 8 years	7 weeks	HP: additionally 1.2 g/kg·day of P supplement (mixture of casein, gelatin, vegetable proteins, yeast, and soy) DCD: 30 g of F (62% SFA and 29% MUFAs).	Not reported	HP decreased from 10.7 ± 3.2 mmol/L to 10.2 ± 3.2 mmol/L * vs. DCD from 10.7 ± 3.3 to 9.3 ± 3.5 *

* *p* ≤ 0.05; ₹ *p* ≤ 0.01; + *p* ≤ 0.001; ¥ *p* ≤ 0.0001; NS: non-significant; HP: high-protein diet; DCD: diabetes conventional diet, PUFA: polyunsaturated fatty acid; SFA: saturated fatty acid; MUFA: monounsaturated fatty acids; HbA1c: glycosylated hemoglobin; FBG: fasting blood glucose; BMI: body mass index; T2DM: type 2 diabetes mellitus; CH: carbohydrates; P: protein; F: fat; NP: not provided.

**Table 3:** Potential beneficial and negative effects of high-protein diets (HPDs).

Beneficial	Negative
Promotes an adequate nutritional status from essential amino acids and protein synthesis [16]	Renal dysfunction by increasing glomerular blood pressure and GFR * [33,95] **
Improves glycemic control by increasing the release of insulin and insulin sensitivity [17,30,31]	Osteoporosis due to accelerated bone resorption and urinary excretion of calcium by renal acid load [33,96]
Decreases body adiposity by increasing resting caloric expenditure [17,18,19]	Metabolic acidosis caused by acid load from animal-based dietary sources [95]
Decreases risk of obesity by improving satiety [20,33,34]	Cardiovascular risk due to the formation of atherosclerotic plaques [97,98]

* GFR, glomerular filtration rate. ** The same sources, however, admit that there is no consensus on this effect, since several studies show no kidney damage by HPDs.

## Data Availability

The original contributions presented in the study are included in the article; further inquiries can be directed to the corresponding author/s.

## Supplementary Materials

The following supporting information can be downloaded at: https://www.mdpi.com/article/10.3390/ijms252010959/s1. Refs. [42,43,44,45,46,47,48,49,50,51,52,53,54,55,56,57,58,59,60,61,62,63,64,65,66,67,68,69,70,71,72,73,74,75,76,77,78,79,80,81] are cited within the supplementary.

## References

[1] American Diabetes Association (2019). 2. Classification and diagnosis of diabetes: Standards of medical care in diabetes. Diabetes Care.

[2] Kerner W., Brückel J. (2014). Definition, classification and diagnosis of diabetes mellitus. Exp. Clin. Endocrinol. Diabetes.

[3] International Diabetes Federation Type 2 Diabetes. https://www.idf.org/aboutdiabetes/type-2-diabetes.html.

[4] IDF Diabetes Atlas (2021). Diabetes around the World in 2021. https://diabetesatlas.org/.

[5] Myles I.A. (2014). Fast food fever: Reviewing the impacts of the Western diet on immunity. Nutr. J..

[6] Briggs Early K., Stanley K. (2018). Position of the Academy of Nutrition and Dietetics: The Role of Medical Nutrition and Therapy and Registered Dietitian Nutritionists in the Prevention and Treatment of Prediabetes and Type 2 Diabetes. J. Acad. Nutr. Diet..

[7] American Diabetes Association (2019). 5. Lifestyle management: Standards of medical care in diabetes. Diabetes Care.

[8] American Diatebes Association (ADA) What is the Diabetes Plate?. 2020..

[9] Riobó Serván P. (2018). Pautas dietéticas en la diabetes y en la obesidad. Nutr. Hosp..

[10] Rodríguez Amador L., Carlos Sosa Pérez J., Fidel Buchaca Faxas E., Fernández Valdés F., Antonio Bermúdez Rojas S., Mora I. (2015). Niveles de hemoglobina glucosilada y su correlación con las glucemias de ayuno y postprandial en un grupo de pacientes diabéticos. Acta Médica de Cuba.

[11] American Diabetes Association (2019). 6. Glycemic targets: Standards of medical care in diabetes. Diabetes Care.

[12] Choi S.W., Friso S. (2010). Epigenetics: A New Bridge between Nutrition and Health. Adv. Nutr..

[13] Napoli C., Benincasa G., Schiano C., Salvatore M. (2020). Differential epigenetic factors in the prediction of cardiovascular risk in diabetic patients. Eur. Heart J. Cardiovasc. Pharmacother..

[14] Lorenzo P.M., Izquierdo A.G., Rodriguez-Carnero G., Fernández-Pombo A., Iglesias A., Carreira M.C., Tejera C., Bellido D., Martinez-Olmos M.A., Leis R. (2022). Epigenetic Effects of Healthy Foods and Lifestyle Habits from the Southern European Atlantic Diet Pattern: A Narrative Review. Adv. Nutr..

[15] Abubakar B., Usman D., Sanusi K.O., Azmi N.H., Imam M.U. (2023). Preventive Epigenetic Mechanisms of Functional Foods for Type 2 Diabetes. Diabetology.

[16] Gonzáles G.F., Villena A. (2002). Obesidad: Dietas Hipocalóricas y el Suplemento con Aminoácidos. Rev. Peru. De Endocrinol. Y Metab..

[17] Adlersberg D. (1948). The use of high protein diets in the treatment of diabetes mellitus. Am. J. Dig. Dis..

[18] Giglio B.M., Duarte V.I., Galvão A.F., Marini A.C.B., Schincaglia R.M., Mota J.F., Souza L.B., Pimentel G.D. (2019). High-protein diet containing dairy products is associated with low body mass index and glucose concentrations: A cross-sectional study. Nutrients.

[19] Nuttall F.Q., Gannon M.C. (2004). Metabolic response of people with type 2 diabetes to a high protein diet. Nutr. Metab..

[20] Flakoll P.J., Kulaylat M., Frexes-Steed M., Hill J.O., Abumrad N.N. (1991). Amino acids enhance insulin resistance to exogenous glucose infusion in overnight-fasted humans. J. Parenter. Enter. Nutr..

[21] Westerterp-Plantenga M.S., Lemmens S.G., Westerterp K.R. (2012). Dietary protein—Its role in satiety, energetics, weight loss and health. Br. J. Nutr..

[22] Pfeiffer A.F., Pedersen E., Schwab U., Risérus U., Aas A.M., Uusitupa M., Thanopoulou A., Kendall C., Sievenpiper J.L., Kahleová H. (2020). The effects of different quantities and qualities of protein intake in people with diabetes mellitus. Nutrients.

[23] Bloomgarden Z. (2018). Diabetes and branched-chain amino acids: What is the link?. J. Diabetes.

[24] Arrieta-Cruz I., Su Y., Gutiérrez-Juárez R. (2016). Suppression of Endogenous Glucose Production by Isoleucine and Valine and Impact of Diet Composition. Nutrients.

[25] Yoshizawa F. (2012). New therapeutic strategy for amino acid medicine: Notable functions of branched chain amino acids as biological regulators. J. Pharmacol. Sci..

[26] Kawaguchi T., Nagao Y., Matsuoka H., Ide T., Sata M. (2008). Branched-chain amino acid-enriched supplementation improves insulin resistance in patients with chronic liver disease. Int. J. Mol. Med..

[27] Schwenk W.F., Haymond M.W. (1987). Decreased uptake of glucose by human forearm during infusion of leucine, isoleucine, or threonine. Diabetes.

[28] Yang J., Chi Y., Burkhardt B.R., Guan Y., Wolf B.A. (2010). Leucine metabolism in regulation of insulin secretion from pancreatic beta cells. Nutr. Rev..

[29] Nagata C., Nakamura K., Wada K., Tsuji M., Tamai Y., Kawachi T. (2013). Branched-chain amino acid intake and the risk of diabetes in a Japanese community: The Takayama study. Am. J. Epidemiol..

[30] Su Y., Lam T.K., He W., Pocai A., Bryan J., Aguilar-Bryan L., Gutiérrez-Juárez R. (2012). Hypothalamic leucine metabolism regulates liver glucose production. Diabetes.

[31] Arrieta-Cruz I., Gutiérrez-Juárez R. (2016). The Role of Circulating Amino Acids in the Hypothalamic Regulation of Liver Glucose Metabolism. Adv. Nutr..

[32] Stentz F.B., Mikhael A., Kineish O., Christman J., Sands C. (2021). High protein diet leads to prediabetes remission and positive changes in incretins and cardiovascular risk factors. Nutr. Metab. Cardiovasc. Dis..

[33] Moon J., Koh G. (2020). Clinical Evidence and Mechanisms of High-Protein Diet-Induced Weight Loss. J. Obes. Metab. Syndr..

[34] Yu D., Richardson N.E., Green C.L., Spicer A.B., Murphy M.E., Flores V., Jang C., Kasza I., Nikodemova M., Wakai M.H. (2021). The adverse metabolic effects of branched-chain amino acids are mediated by isoleucine and valine. Cell Metab..

[35] Avery C.L., Howard A.G., Lee H.H., Downie C.G., Lee M.P., Koenigsberg S.H., Ballou A.F., Preuss M.H., Raffield L.M., Yarosh R.A. (2023). Branched chain amino acids harbor distinct and often opposing effects on health and disease. Commun. Med..

[36] Green C.L., Trautman M.E., Chaiyakul K., Jain R., Alam Y.H., Babygirija R., Pak H.H., Sonsalla M.M., Calubag M.F., Yeh C.Y. (2023). Dietary restriction of isoleucine increases healthspan and lifespan of genetically heterogeneous mice. Cell Metab..

[37] Neinast M., Murashige D., Arany Z. (2019). Branched Chain Amino Acids. Annu. Rev. Physiol..

[38] Wang T.J., Larson M.G., Vasan R.S., Cheng S., Rhee E.P., McCabe E., Lewis G.D., Fox C.S., Jacques P.F., Fernandez C. (2011). Metabolite profiles and the risk of developing diabetes. Nat. Med..

[39] White P.J., McGarrah R.W., Herman M.A., Bain J.R., Shah S.H., Newgard C.B. (2021). Insulin action, type 2 diabetes, and branched-chain amino acids: A two-way street. Mol. Metab..

[40] Sarkis-Onofre R., Catalá-López F., Aromataris E., Lockwood C. (2021). How to properly use the PRISMA Statement. Syst. Rev..

[41] The Cochrane Collaboration (2020). Review Manager (RevMan).

[42] Azwan K., Mona R., Firdous J., Sari D.K., David P.R., Muhammad N. (2021). Low weight gain, better glycaemia and no central obesity achieved through a high-protein diet. J. Med. Pharm. Allied Sci..

[43] Huang G., Pencina K., Li Z., Apovian C.M., Travison T.G., Storer T.W., Gagliano-Jucá T., Basaria S., Bhasin S. (2021). Effect of Protein Intake on Visceral Abdominal Fat and Metabolic Biomarkers in Older Men with Functional Limitations: Results From a Randomized Clinical Trial. J. Gerontol. A Biol. Sci. Med. Sci..

[44] Tettamanzi F., Bagnardi V., Louca P., Nogal A., Monti G.S., Mambrini S.P., Lucchetti E., Maestrini S., Mazza S., Rodriguez-Mateos A. (2021). A High Protein Diet Is More Effective in Improving Insulin Resistance and Glycemic Variability Compared to a Mediterranean Diet-A Cross-Over Controlled Inpatient Dietary Study. Nutrients.

[45] Thomsen M.N., Skytte M.J., Astrup A., Deacon C.F., Holst J.J., Madsbad S., Krarup T., Haugaard S.B., Samkani A. (2020). The clinical effects of a carbohydrate-reduced high-protein diet on glycaemic variability in metformin-treated patients with type 2 diabetes mellitus: A randomised controlled study. Clin. Nutr. ESPEN.

[46] de Luis D.A., Izaola O., Primo D., Aller R. (2020). A circadian rhythm-related MTNR1B genetic variant (rs10830963) modulate body weight change and insulin resistance after 9 months of a high protein/low carbohydrate vs a standard hypocaloric diet. J. Diabetes Its Complicat..

[47] Skytte M.J., Samkani A., Astrup A., Larsen T.M., Frystyk J., Poulsen H.E., Henriksen T., Holst J.J., Andersen O., Madsbad S. (2020). Effects of a highly controlled carbohydrate-reduced high-protein diet on markers of oxidatively generated nucleic acid modifications and inflammation in weight stable participants with type 2 diabetes; a randomized controlled trial. Scandinavian J. Clin. Lab. Investig..

[48] Hjorth M.F., Bray G.A., Zohar Y., Urban L., Miketinas D.C., Williamson D.A., Ryan D.H., Rood J., Champagne C.M., Sacks F.M. (2019). Pretreatment Fasting Glucose and Insulin as Determinants of Weight Loss on Diets Varying in Macronutrients and Dietary Fibers-The POUNDS LOST Study. Nutrients.

[49] Winn N.C., Pettit-Mee R., Walsh L.K., Restaino R.M., Ready S.T., Padilla J., Kanaley J.A. (2019). Metabolic Implications of Diet and Energy Intake during Physical Inactivity. Med Sci Sports Exerc..

[50] Li J., Polston K.F.L., Eraslan M., Bickel C.S., Windham S.T., McLain A.B., Oster R.A., Bamman M.M., Yarar-Fisher C.A. (2018). high-protein diet or combination exercise training to improve metabolic health in individuals with long-standing spinal cord injury: A pilot randomized study. Physiol. Rep..

[51] Shan R., Duan W., Liu L., Qi J., Gao J., Zhang Y., Du S., Han T., Pang X., Sun C. (2018). Low-Carbohydrate, High-Protein, High-Fat Diets Rich in Livestock, Poultry and Their Products Predict Impending Risk of Type 2 Diabetes in Chinese Individuals that Exceed Their Calculated Caloric Requirement. Nutrients.

[52] Johnston C.S., Sears B., Perry M., Knurick J.R. (2017). Use of Novel High-Protein Functional Food Products as Part of a Calorie-Restricted Diet to Reduce Insulin Resistance and Increase Lean Body Mass in Adults: A Randomized Controlled Trial. Nutrients.

[53] Mateo-Gallego R., Marco-Benedí V., Perez-Calahorra S., Bea A.M., Baila-Rueda L., Lamiquiz-Moneo I., de Castro-Orós I., Cenarro A., Civeira F. (2017). Energy-restricted, high-protein diets more effectively impact cardiometabolic profile in overweight and obese women than lower-protein diets. Clin. Nutr..

[54] Smith G.I., Yoshino J., Kelly S.C., Reeds D.N., Okunade A., Patterson B.W., Klein S., Mittendorfer B. (2016). High-Protein Intake during Weight Loss Therapy Eliminates the Weight-Loss-Induced Improvement in Insulin Action in Obese Postmenopausal Women. Cell Rep..

[55] De Luis D.A., Aller R., Izaola O., Reeds D.N., Okunade A., Patterson B.W., Klein S., Mittendorfer B. (2015). Effects of a High-Protein/Low-Carbohydrate versus a Standard Hypocaloric Diet on Weight and Cardiovascular Risk Factors during 9 Months: Role of a Genetic Variation in the Cannabinoid Receptor Gene (CNR1) (G1359A Polymorphism). Ann. Nutr. Metab..

[56] Qi Q., Zheng Y., Huang T., Rood J., Bray G.A., Sacks F.M., Qi L. (2015). Vitamin D metabolism-related genetic variants, dietary protein intake and improvement of insulin resistance in a 2 year weight-loss trial: POUNDS Lost. Diabetologia.

[57] Tay J., Thompson C.H., Luscombe-Marsh N.D., Noakes M., Buckley J.D., Wittert G.A., Brinkworth G.D. (2015). Long-Term Effects of a Very Low Carbohydrate Compared with a High Carbohydrate Diet on Renal Function in Individuals with Type 2 Diabetes: A Randomized Trial. Medicine.

[58] Pedersen E., Jesudason D.R., Clifton P.M. (2014). High protein weight loss diets in obese subjects with type 2 diabetes mellitus. Nutr. Metab. Cardiovasc. Dis..

[59] Von Bibra H., Wulf G., St John Sutton M., Pfützner A., Schuster T., Heilmeyer P. (2014). Low-carbohydrate/high-protein diet improves diastolic cardiac function and the metabolic syndrome in overweight-obese patients with type 2 diabetes. IJC Metab. Endocr..

[60] Chen H.Y., Zhou J., Wang Z., Li Y.Y., Li M.L. (2013). Effects of normal caloric high-protein diet on metabolic parameters and gastrointestinal hormones in patients with obesity or type 2 diabetes mellitus. Chin. J. Clin. Nutr..

[61] Luger M., Holstein B., Schindler K., Kruschitz R., Ludvik B. (2013). Feasibility and efficacy of an isocaloric high-protein vsstandard diet on insulin requirement, body weight and metabolic parameters in patients with type 2 diabetes on insulin therapy. Exp. Clin. Endocrinol. Diabetes.

[62] Rizkalla S.W., Prifti E., Cotillard A., Pelloux V., Rouault C., Allouche R., Laromiguiere M., Kong L., Darakhshan F., Massiera F. (2012). Differential effects of macronutrient content in 2 energy-restricted diets on cardiovascular risk factors and adipose tissue cell size in moderately obese individuals: A randomized controlled trial. Am. J. Clin. Nutr..

[63] Bortolotti M., Maiolo E., Corazza M., Van Dijke E., Schneiter P., Boss A., Carrel G., Giusti V., Lê K.A., Chong D.G.Q. (2011). Effects of a whey protein supplementation on intrahepatocellular lipids in obese female patients. Clin. Nutr..

[64] Kreider R.B., Rasmussen C., Kerksick C.M., Wilborn C., Taylor L., Campbell B., Magrans-Courtney T., Fogt D., Ferreira M., Li R. (2011). A carbohydrate-restricted diet during resistance training promotes more favorable changes in body composition and markers of health in obese women with and without insulin resistance. Phys. Sportsmed..

[65] Pearce K.L., Clifton P.M., Noakes M. (2011). Egg consumption as part of an energy-restricted high-protein diet improves blood lipid and blood glucose profiles in individuals with type 2 diabetes. Br. J. Nutr..

[66] Te Morenga L.A., Levers M.T., Williams S.M., Brown R.C., Mann J. (2011). Comparison of high protein and high fiber weight-loss diets in women with risk factors for the metabolic syndrome: A randomized trial. Nutr. J..

[67] Farnsworth E., Luscombe N.D., Noakes M., Wittert G., Argyiou E., Clifton P.M. (2003). Effect of a high-protein, energy-restricted diet on body composition, glycemic control, and lipid concentrations in overweight and obese hyperinsulinemic men and women. Am. J. Clin. Nutr..

[68] Nuttall F.Q., Gannon M.C., Saeed A., Jordan K., Hoover H. (2003). The metabolic response of subjects with type 2 diabetes to a high-protein, weight-maintenance diet. J. Clin. Endocrinol. Metab..

[69] Piatti P.M., Monti L.D., Magni F., Fermo I., Baruffaldi L., Nasser R., Santambrogio G., Librenti M.C., Galli-Kienle M., Pontiroli A.E. (1994). Hypocaloric high-protein diet improves glucose oxidation and spares lean body mass: Comparison to hypocaloric high-carbohydrate diet. Metabolism.

[70] Ireland P., O’Dea K., Nankervis A. (1992). Short-term effects of alterations in dietary fat on metabolic control in IDDM. Diabetes Care.

[71] Fitz J.D., Sperling E.M., Fein H.G. (1983). A hypocaloric high-protein diet as primary therapy for adults with obesity-related diabetes: Effective long-term use in a community hospital. Diabetes Care.

[72] Thomsen M.N., Skytte M.J., Samkani A., Carl M.H., Weber P., Astrup A., Chabanova E., Fenger M., Frystyk J., Hartmann B. (2022). Dietary carbohydrate restriction augments weight loss-induced improvements in glycaemic control and liver fat in individuals with type 2 diabetes: A randomised controlled trial. Diabetologia..

[73] Skytte M.J., Samkani A., Petersen A.D., Thomsen M.N., Astrup A., Chabanova E., Frystyk J., Holst J.J., Thomsen H.S., Madsbad S. (2019). A carbohydrate-reduced high-protein diet improves HbA1c and liver fat content in weight stable participants with type 2 diabetes: A randomised controlled trial. Diabetologia.

[74] Liu K., Wang B., Zhou R., Lang H.D., Ran L., Wang J., Li L., Kang C., Zhu X.H., Zhang Q.Y. (2018). Effect of combined use of a low-carbohydrate, high-protein diet with omega-3 polyunsaturated fatty acid supplementation on glycemic control in newly diagnosed type 2 diabetes: A randomized, double-blind, parallel-controlled trial. Am. J. Clin. Nutr..

[75] Samkani A., Skytte M.J., Kandel D., Kjaer S., Astrup A., Deacon C.F., Holst J.J., Madsbad S., Rehfeld J.F., Haugaard S.B. (2018). A carbohydrate-reduced high-protein diet acutely decreases postprandial and diurnal glucose excursions in type 2 diabetes patients. Br. J. Nutr..

[76] Wycherley T.P., Noakes M., Clifton P.M., Cleanthous X., Keogh J.B., Brinkworth G.D. (2010). A high-protein diet with resistance exercise training improves weight loss and body composition in overweight and obese patients with type 2 diabetes. Diabetes Care.

[77] Nuttall F.Q., Schweim K., Hoover H., Gannon M.C. (2008). Effect of the LoBAG30 diet on blood glucose control in people with type 2 diabetes. Br. J. Nutr..

[78] McAuley K.A., Smith K.J., Taylor R.W., McLay R.T., Williams S.M., Mann J.I. (2006). Long-term effects of popular dietary approaches on weight loss and features of insulin resistance. Int. J. Obes..

[79] Sargrad K.R., Homko C., Mozzoli M., Boden G. (2005). Effect of high protein vs high carbohydrate intake on insulin sensitivity, body weight, hemoglobin A1c, and blood pressure in patients with type 2 diabetes mellitus. J. Am. Diet. Assoc..

[80] Gannon M.C., Nuttall F.Q., Saeed A., Jordan K., Hoover H. (2003). An increase in dietary protein improves the blood glucose response in persons with type 2 diabetes. Am. J. Clin. Nutr..

[81] Pomerleau J., Verdy M., Garrel D.R., Nadeau M.H. (1993). Effect of Protein Intake on Glycaemic Control and Renal Function in Type 2 (Non-Insulin-Dependent) Diabetes Mellitus. Diabetologia.

[82] Zeevi D., Korem T., Zmora N., Israeli D., Rothschild D., Weinberger A., Ben-Yacov O., Lador D., Avnit-Sagi T., Lotan-Pompan M. (2015). Personalized Nutrition by Prediction of Glycemic Responses. Cell.

[83] Boutron I., Page M.J., Higgins J.P.T., Altman D.G., Lundh A., Hróbjartsson A., Higgins J.P.T., Thomas J., Chandler J., Cumpston M., Li T., Page M.J., Welch V.A. (2024). Chapter 7: Considering bias and conflicts of interest among the included studies [last updated August 2022]. Cochrane Handbook for Systematic Reviews of Interventions Version 6.5.

[84] Wickstrom G., Bendix T. (2000). The “Hawthorne effect”—What did the original Hawthorne studies actually show?. Scand. J. Work Environ. Health.

[85] Markova M., Pivovarova O., Hornemann S., Sucher S., Frahnow T., Wegner K., Machann J., Petzke K.J., Hierholzer J., Lichtinghagen R. (2017). Isocaloric Diets High in Animal or Plant Protein Reduce Liver Fat and Inflammation in Individuals with Type 2 Diabetes. Gastroenterology.

[86] Mariotti F. (2019). Animal and Plant Protein Sources and Cardiometabolic Health. Adv. Nutr..

[87] Glass C.K., Olefsky J.M. (2012). Inflammation and lipid signaling in the etiology of insulin resistance. Cell Metab..

[88] Forouhi N.G., Misra A., Mohan V., Taylor R., Yancy W. (2018). Science and Politics of Nutrition: Dietary and nutritional approaches for prevention and management of type 2 diabetes. BMJ.

[89] Jönsson T., Granfeldt Y., Ahrén B., Branell U.C., Pålsson G., Hansson A., Söderström M., Lindeberg S. (2009). Beneficial effects of a Paleolithic diet on cardiovascular risk factors in type 2 diabetes: A randomized cross-over pilot study. Cardiovasc. Diabetol..

[90] Masharani U., Sherchan P., Schloetter M., Stratford S., Xiao A., Sebastian A., Nolte Kennedy M., Frassetto L. (2015). Metabolic and physiologic effects from consuming a hunter-gatherer (Paleolithic)-type diet in type 2 diabetes. Eur. J. Clin. Nutr..

[91] Eaton S.B., Konner M. (1985). Paleolithic Nutrition. N. Engl. J. Med..

[92] Fontes-Villalba M., Lindeberg S., Granfeldt Y., Knop F.K., Memon A.A., Carrera-Bastos P., Picazo Ó., Chanrai M., Sunquist J., Sundquist K. (2016). Palaeolithic diet decreases fasting plasma leptin concentrations more than a diabetes diet in patients with type 2 diabetes: A randomised cross-over trial. Cardiovasc. Diabetol..

[93] Watanabe S. (2017). Low-protein diet for the prevention of renal failure. Proc. Jpn. Acad. Ser. B Phys. Biol. Sci..

[94] Tanaka S., Wakui H., Azushima K., Tsukamoto S., Yamaji T., Urate S., Suzuki T., Abe E., Taguchi S., Yamada T. (2023). Effects of a High-Protein Diet on Kidney Injury under Conditions of Non-CKD or CKD in Mice. Int. J. Mol. Sci..

[95] Ko G.J., Rhee C.M., Kalantar-Zadeh K., Joshi S. (2020). The Effects of High-Protein Diets on Kidney Health and Longevity. J. Am. Soc. Nephrol..

[96] Cao J.J., Nielsen F.H. (2010). Acid diet (high-meat protein) effects on calcium metabolism and bone health. Curr. Opin. Clin. Nutr. Metab. Care.

[97] Zhang X., Sergin I., Evans T.D., Jeong S.J., Rodriguez-Velez A., Kapoor D., Chen S., Song E., Holloway K.B., Crowley J.R. (2020). High-protein diets increase cardiovascular risk by activating macrophage mTOR to suppress mitophagy. Nat. Metab..

[98] Ge L., Sadeghirad B., Ball G.D.C., da Costa B.R., Hitchcock C.L., Svendrovski A., Kiflen R., Quadri K., Kwon H.Y., Karamouzian M. (2020). Comparison of dietary macronutrient patterns of 14 popular named dietary programmes for weight and cardiovascular risk factor reduction in adults: Systematic review and network meta-analysis of randomised trials. BMJ.

