# Supplementary material for: Efficacy of a High-Protein Diet to Lower Glycemic Levels in Type 2 Diabetes Mellitus: A Systematic Review
Source: Int J Mol Sci. 2024 Oct 11;25(20):10959. doi: 10.3390/ijms252010959 (PMC11507302; doi:10.3390/ijms252010959)
Supplement: Supplementary file 1 [file ijms-25-10959-s001.zip › ijms-3140659-supplementary.pdf]

Table S1. Articles not included in the study and reason(s) why they were excluded.

Study	Reason why it was excluded
**Azwan 2021** [42]	The study was carried out in rats
**Huang 2021** [43]	The participants did not have T2DM
**Tettamanzi 2021** [44]	The comparison was not made with a DCD
**Thomsen 2020** [45]	The study did not have FBG or HbA1c results
**De Luis 2020** [46]	The study used hypocaloric diets
**Skytte 2020** [47]	The study did not have FBG or HbA1c results
**Hjorth 2019** [48]	The study used hypocaloric diets
**Winn 2019** [49]	The study used hypocaloric diets
**Li 2018** [50]	The participants did not have T2DM
**Shan 2018** [51]	The study had a cross-sectional design
**Johnston 2017** [52]	The participants did not have T2DM
**Mateo-Gallego 2017** [53]	The study used hypocaloric diets
**Smith 2016** [54]	The study used hypocaloric diets
**De Luis 2015** [55]	The study used hypocaloric diets
**Qi 2015** [56]	The study used hypocaloric diets
**Tay 2015** [57]	The study did not have FBG or HbA1c results
**Pedersen 2014** [58]	The study used hypocaloric diets
**Von Bibra 2014** [59]	The study used hypocaloric diets
**Chen 2013** [60]	The study did not have FBG or HbA1c results
**Luger 2013** [61]	The participants used insulin
**Rizkalla 2012** [62]	The study used hypocaloric diets
**Bortolotti 2011** [63]	The study did not have FBG or HbA1c results
**Kreider 2011** [64]	The study used hypocaloric diets
**Pearce 2011** [65]	The comparison was not made with a DCD
**Te morenga 2011** [66]	The participants did not have T2DM
**Farnswort 2003** [67]	The participants did not have T2DM
**Nutall 2003** [68]	The study did not have FBG or HbA1c results
**Piatti 1994** [69]	The study used hypocaloric diets
**Ireland 1992** [70]	The comparison was not made with a DCD
**Fitz 1983** [71]	The participants used insulin

T2DM: type 2 diabetes mellitus, DCD: Diabetes conventional diet, FBG: fasting blood glucose, HbA1c: glycosylated hemoglobin.

Table S2. Body Mass Index (BMI) changes during treatments or between study groups				
**STUDY**	**BMI BASELINE (kg/m²)**	**BMI FINAL (kg/m²)**	**CHANGE**	**BODY WEIGHT (Kg)**
**Thomsen 2022** [72]	HP: 33.2 ± 5.1		−2.0 ± 0.6‡	
	CD: 33.6 ± 4.6		−2.0 ± 0.6‡	
**Skytte 2019** [73]	HP: 29.4 ± 1.0		−0.5 (± 0.1)£	
	CD: 29.4 ± 0.9		−0.3 (± 0.1)£	
**Liu 2018** [74]	18.5 - 23.9		Unchanged (NS)	
**Samkani 2018** [75]	30 ± 4.4	ND	ND	ND
**Wycherley 2010** [76]	HP: 35.6 ± 3.8<br>CD: 34.8 ± 4.9	32.5 ± 3.1<br>31.7 ± 5.1	3.2 ± 1.7‡<br>3.1 ± 1.6‡	
**Nuttall 2008** [77]	24-35	ND	ND	Unchanged
**McAuley 2006** [78]	ND	ND	ND	HP: 0.6 (0.2, 1.0)¥
				CD: 0.4 (-0.0, 0.8)¥
**Sargrard 2005** [79]	36 ± 3	ND	ND	HP: -2.5±1.6¥
				CD: -2.2±0.9¥
**Gannon 2003** [80]	31 (22 - 37)	ND	ND	Unchanged
**Pomerleau 1993** [81]	> 27		HP: -1.0 ± 0.9 (-1%)€	
			CD: -1.9 ± 1.5 (-2%)‡	

NS, non-significant; ‡: p<0.001; £: p=0.070 ; ¥: p<0.05 ; €: p=0.004; ND, not determined. NP, not provided.
